# A case report of femoral head fracture with osteochondral lesion treated by osteosynthesis and biomimetic scaffold: 2-year clinical and radiological follow-up

**DOI:** 10.1186/s40634-021-00362-x

**Published:** 2021-07-01

**Authors:** Alessandro Casiraghi, Claudio Galante, Marco Domenicucci, Stefano Cattaneo, Andrea Achille Spreafico, Marcello Motta, Paolo Capitani, Giuseppe Milano

**Affiliations:** 1 Department of Bone and Joint Surgery, ASST Spedali Civili, Piazzale Spedali Civili 1, 25123, Brescia (BS), Italy; 2 Residency Program in Orthopedics and Traumatology, University of Milan, Via Festa del Perdono 7, 20122 Milan (MI), Italy; 3 Department of Medical and Surgical Specialties, Radiological Sciences, and Public Health, University of Brescia, Viale Europa 11, 25123 Brescia (BS), Italy; 4 Department of Bone and Joint Surgery, ASST Grande Ospedale Metropolitano Niguarda, Piazza dell’Ospedale Maggiore 3, 20162, Milan (MI), Italy

**Keywords:** Femur head, Fracture, Hip dislocation, Osteochondral lesion, Scaffold

## Abstract

The aim of the present study was to present clinical and radiological outcome of a hip fracture-dislocation of the femoral head treated with biomimetic osteochondral scaffold.

An 18-year-old male was admitted to the hospital after a motorcycle-accident. He presented with an obturator hip dislocation with a type IVA femoral head fracture according to Brumback classification system. The patient underwent surgery 5 days after accident. The largest osteochondral fragment was reduced and stabilized with 2 screws, and the small fragments were removed. The residual osteochondral area was replaced by a biomimetic nanostructured osteochondral scaffold. At 1-year follow-up the patient did not complain of hip pain and could walk without limp. At 2-year follow-up he was able to run with no pain and he returned to practice sports. Repeated radiographs and magnetic resonance imaging studies of the hip showed no signs of osteoarthritis or evidence of avascular necrosis. A hyaline-like signal on the surface of the scaffold was observed with restoration of the articular surface and progressive decrease of the subchondral edema.

The results of the present study showed that the biomimetic nanostructured osteochondral scaffold could be a promising and safe option for the treatment of traumatic osteochondral lesions of the femoral head.

*Study Design:* Case report.

## Introduction

Femoral head fractures constitute a rare injury compared to other proximal femur or hip joint lesions [1]. Due to the intrinsic anatomic stability of the hip, most of these injuries result from high-energy trauma. Approximately two thirds of patients are young adults and associated injuries are extremely common [2]. Femoral head fractures may present in various patterns with or without associated fractures around the hip [3]. Treatment may range from closed reduction to joint replacement, including other options like open reduction and internal fixation, excision of the bony fragment or a valgus hip osteotomy [1, 4].

The amount of damage to the articular cartilage of the femoral head is the main factor affecting the outcome of a femoral head fracture after traumatic hip dislocation [2]. Cartilage injury in the hip joint is challenging due to the difficulties in surgical repair or reconstruction [5]. A variety of techniques have been reported for the treatment of symptomatic chondral and osteochondral lesions; these techniques have shown satisfactory results in other joints; however, the literature regarding the hip joint is limited [5].

Biomimetic three-dimensional acellular scaffolds offered promising results for the treatment of osteochondral lesions in the knee, [6] but there are no data about their efficacy in the treatment of osteochondral lesions of the femoral head.

In the present study we present the clinical and radiological outcome of a traumatic osteochondral lesion of the femoral head treated with a biomimetic nanostructured osteochondral scaffold in a young male patient.

## Case report

**Fig. 1:**
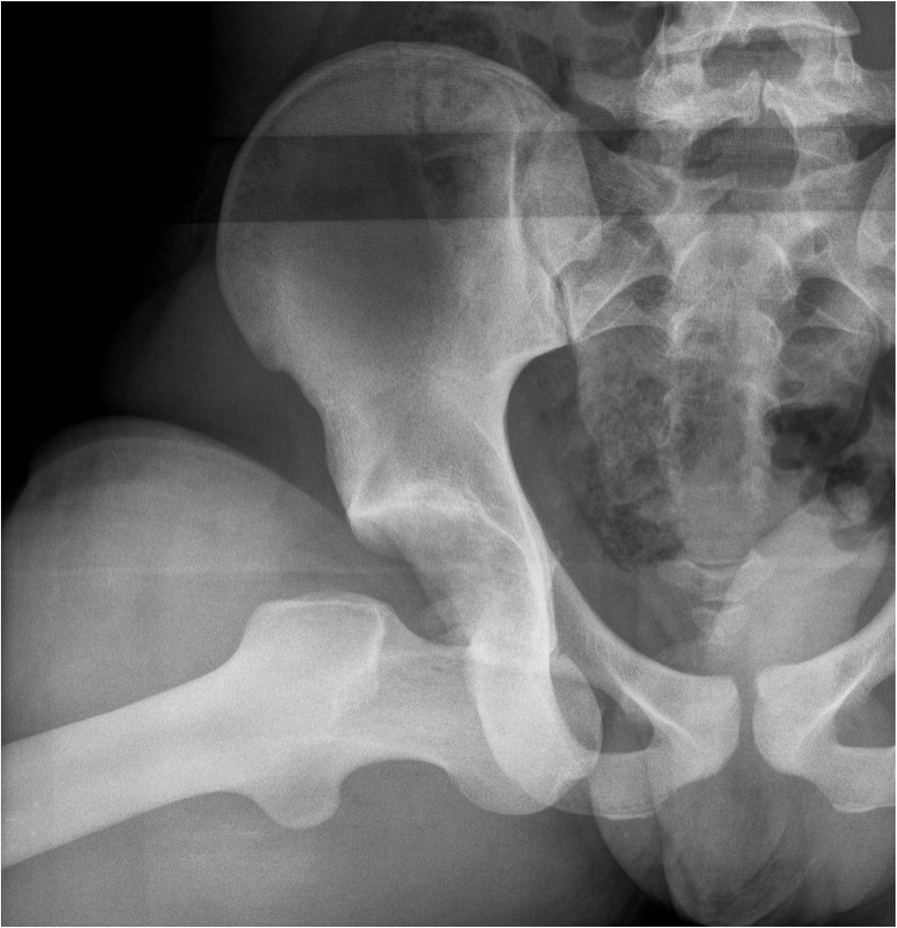
Pelvic radiograph picture showing an obturator hip dislocation with a marginal fracture of the femoral head and an associated severe impaction area on a weight-bearing region

**Fig. 2:**
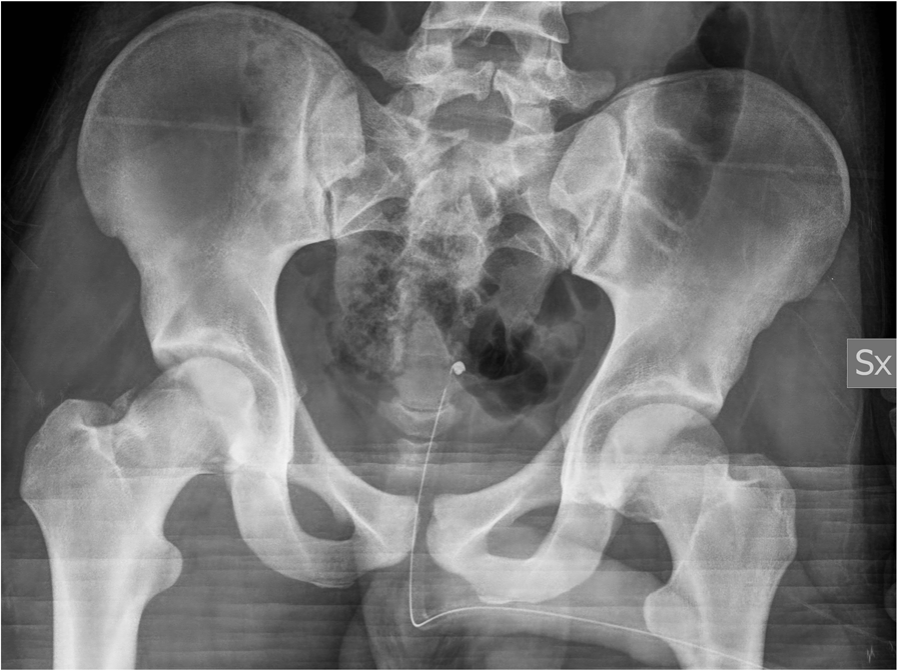
Pelvic radiograph picture showing the closed reduction of the hip joint. The fracture of the femoral head is clearly visible

**Fig. 3:**
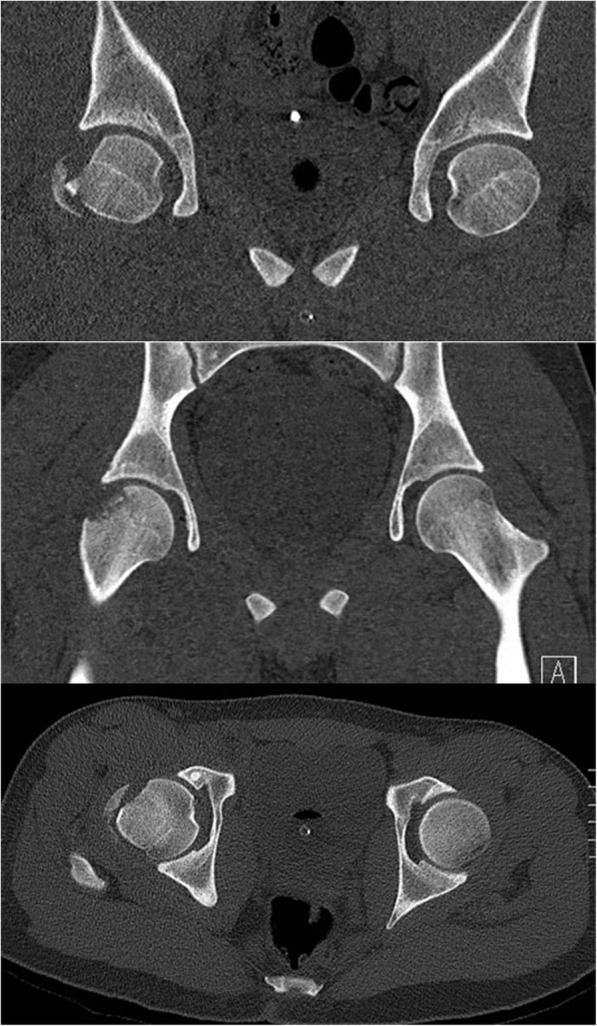
CT scan pictures showing the closed reduction of the hip joint. A type IVA femoral head fracture according to Brumback classification system involving the antero-superior aspect of the femoral head and few fragments displaced anteriorly are visible

An 18-year-old male was admitted to our hospital after a motorcycle accident. He presented with the right lower limb flexed and externally rotated. A pelvic radiograph showed an obturator hip dislocation with a fracture of the femoral head and an intra-articular bony fragment (Fig. 1). He was also diagnosed with non-concussive head injury and splenic contusion. At the emergency department a closed reduction of the hip joint was performed in general anesthesia (Fig. 2). A CT scan after reduction showed a type IVA femoral head fracture according to Brumback classification system [7] that involved the anterosuperior aspect of the femoral head in a weight-bearing area, with few fragments displaced anteriorly (Fig. 3). No other orthopedic injuries were found.

**Fig. 4:**
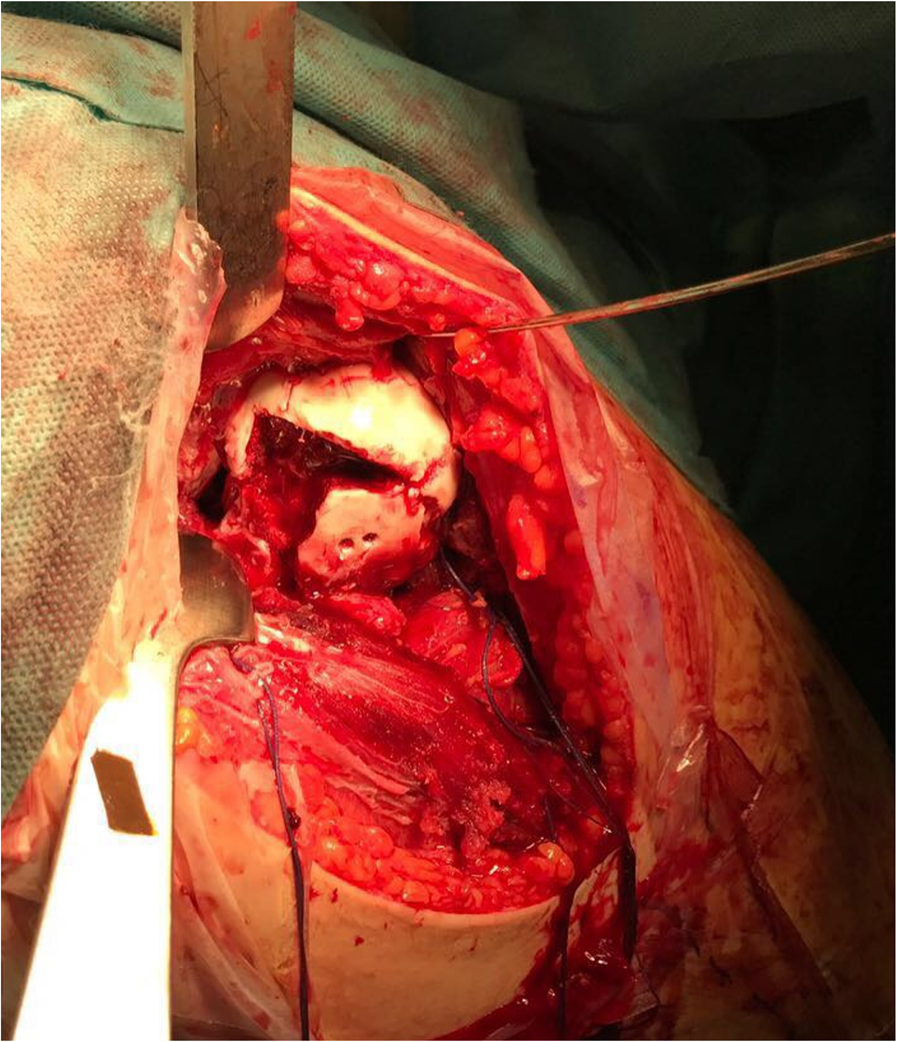
Surgical view: reduction and stabilization of the biggest osteochondral fragment. preparation of the osteochondral biomimetic scaffold lodging

The patient underwent surgical treatment 5 days after accident. He was placed in supine position and an anterior approach with hip dislocation was performed. The biggest osteochondral fragment was reduced and stabilized with 2 screws, the other fragments were too small to allow a stable fixation and were removed (Fig. 4). The residual irregular osteochondral defect area (31mm x 28mm) was filled with a biomimetic nanostructured osteochondral scaffold (MaioRegen; Finceramica Faenza SpA, Faenza, Italy). The MaioRegen osteochondral scaffold consists of a nanostructured biomimetic material with a porous three-dimensional tri-layer composite architecture, mimicking the anatomy of the osteochondral unit [8].

**Fig. 5:**
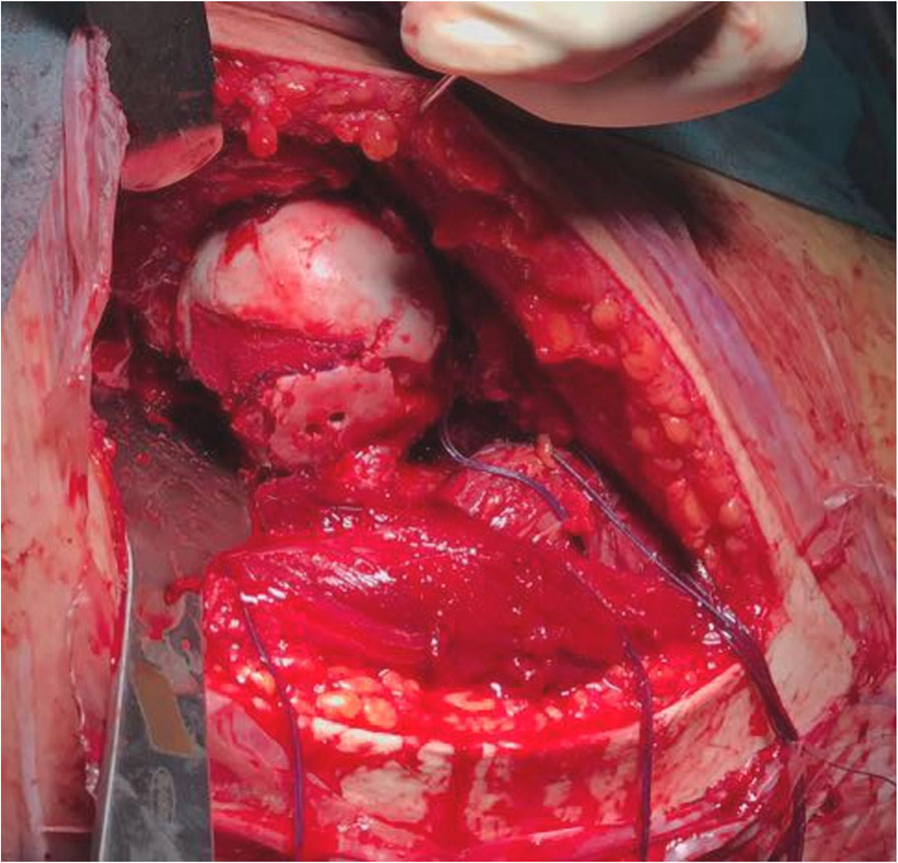
Surgical view: biomimetic osteochondral scaffold implantation. The scaffold has been implanted by press-fit technique. Fibrin glue has been added on the upper edges to ensure post-operative mechanical stability

**Fig. 6:**
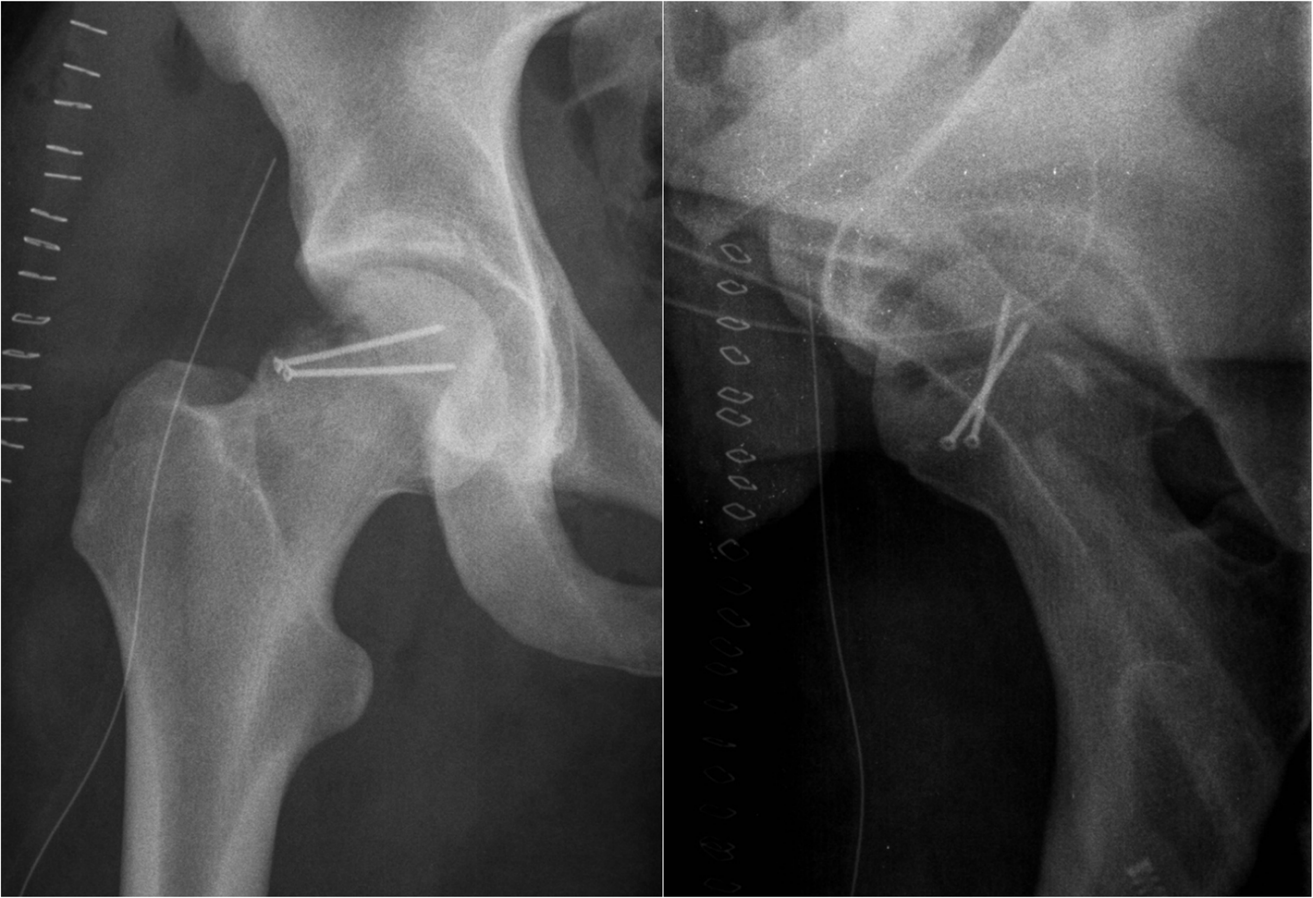
Post-op radiograph picture after reduction of the hip joint, reduction and stabilization of the biggest osteochondral fragment and osteochondral scaffold implantation

Implantation was performed as a single-step surgical procedure [9]. The lesion bed was debrided and flattened with a ring curette and an osteotome up to 8 mm in depth, while leaving perpendicular defect walls. The graft was sized and shaped to obtain optimum fitting into the defects and was then implanted by press-fit technique. Graft was sealed on the upper perimeter with fibrin glue to ensure mechanical stability [10] (Fig. 5). After careful hip reduction, the joint capsule was repaired and the wound was then closed. Postoperative radiographs were obtained to confirm adequate positioning of the scaffold (Fig. 6).

Passive mobilization was allowed immediately after surgery. Weight-bearing was forbidden for 8 weeks in order to avoid mechanical stress to the repair site. After this period, a progressive weight-bearing, cycling and swimming were allowed. Full weight bearing without crutches was restored 16 weeks after surgery. Follow-up visits and radiographs were performed every month until 6 months, then at one and two years after surgery. The patient was also evaluated at 6, 12 and 24 months with magnetic resonance imaging (MRI) exams.

At one-year follow-up the patient did not complain of hip pain and he returned to walk without limp. On clinical examination he had a normal gait and an extension/flexion of 20/0/110, abduction/adduction of 40/0/20 and internal/external rotation of 20/0/40.

At two-year follow-up the patient was able to run without pain and he returned to practice sports.

**Fig. 7:**
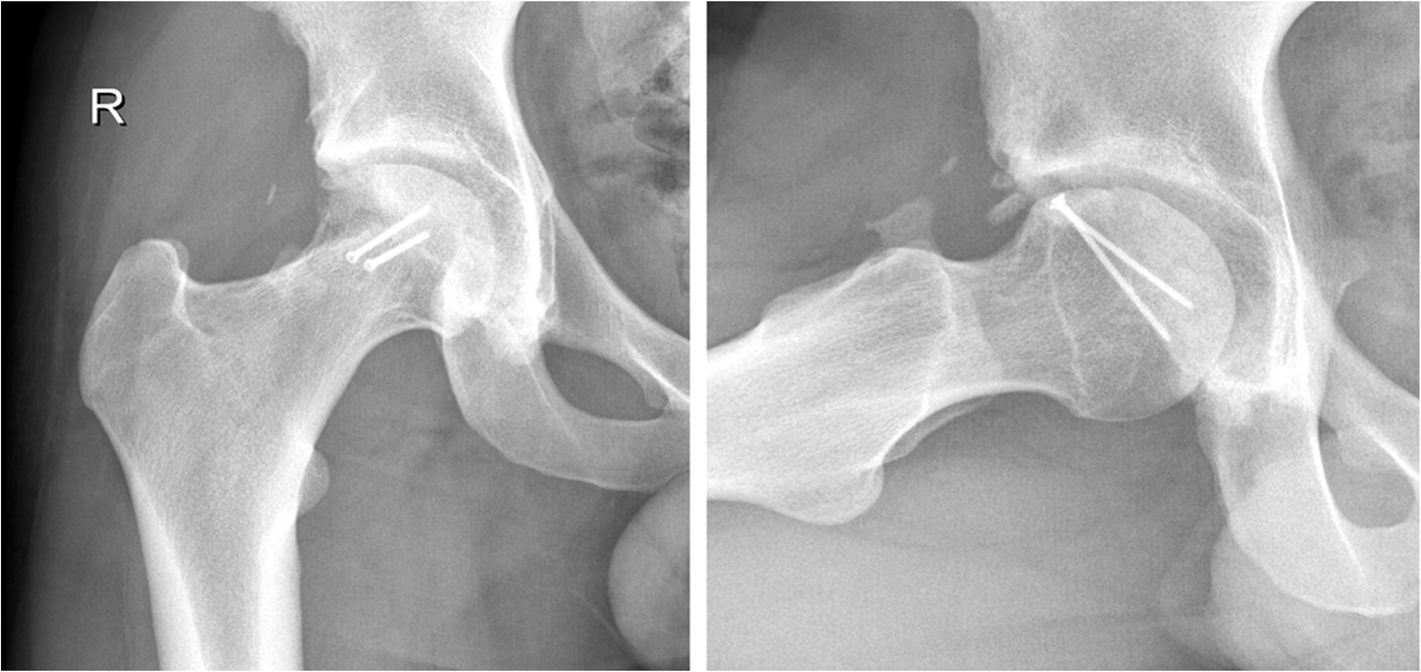
Twenty-four-month follow-up radiograph pictures shows good integration of the stabilized osteochondral fragment and no signs of osteoarthritis or evidence of avascular necrosis

Radiographs obtained at the last follow-up visit showed that the head fracture was completely healed; moreover, there were no signs of osteoarthritis or evidence of avascular necrosis (Fig. 7).

**Fig. 8:**
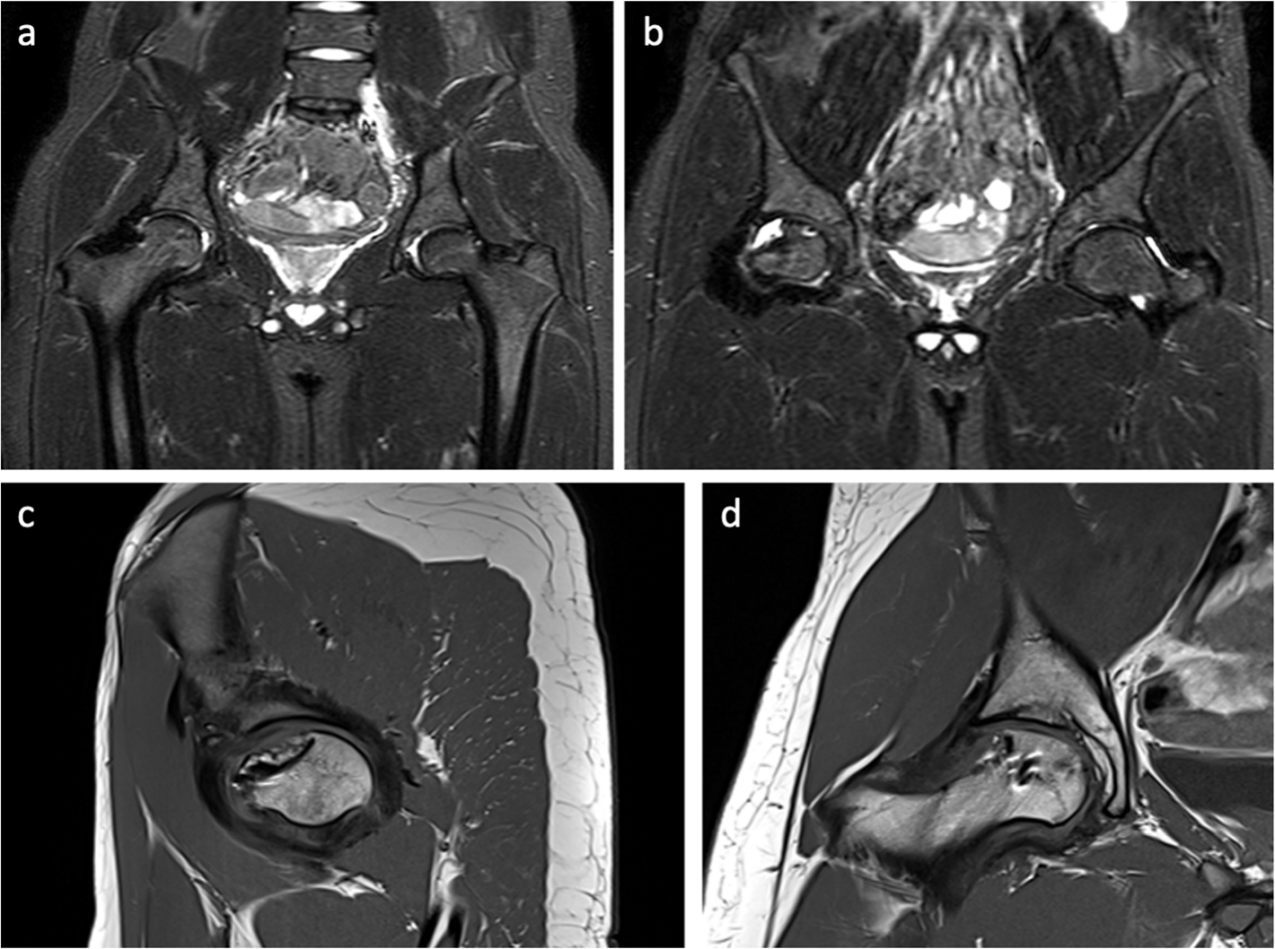
(**a**, **b**) coronal T2 weighted images at 6 months follow-up. The MRI pictures show no sign of bone edema. (**c**, **d**) sagittal and coronal T1 weighted images at 24 months follow-up. The MRI pictures show signs of osteochondral tissue restoration

MRI exams showed that the implant remained in site. At 24 months, a hyaline-like signal with restoration of the articular surface was evident. Subchondral edema progressively decreased with time and at 24 months was barely evident (Fig. 8).

## Discussion

Femoral head fracture, which is mostly caused by high-energy trauma, constitutes a rare injury compared to other proximal femur or hip joint injuries. Nonsurgical treatment of a femoral head fracture is indicated when anatomic reduction is achieved and the hip joint is stable, or if the fracture is inferior to the fovea. Conservative option is unsuitable when the fracture extends above the femoral head fovea into the weight-bearing region [3].

Operative treatment of displaced femoral head fractures can consist of internal fixation or simple excision of the bony fragments. Although some past reports showed similar outcomes between these techniques, [11] a more recent study demonstrated better results with anatomical reduction and internal fixation [12]. Factors that influence treatment choice include fragment size, degree of comminution, and location of the fragment in relation to the weight-bearing surface of the femoral head [3].

As far as the treatment of chondral and osteochondral lesions of the femoral head, a variety of techniques have been proposed [5]. Bone marrow stimulation procedures, such as microfractures, abrasion or drilling, do not only produce a fibrous and softer tissue with respect to native cartilage, with predominance of type I collagen and unorganized extracellular matrix, but they also do not reconstitute normal bone structure, thus further impairing outcomes [13]. Other surgical approachs are the osteochondral autograft implant and the mosaicplasty. Despite the good results even at medium–long-term follow-up, the weakness of this treatments mainly consists of the technical difficulty to restore the physiological curvature of the surface and to obtain plug stability, especially in wide or deep lesions [14]. Moreover, graft availability limits the indication for large lesions due to harvest site morbidity [15]. Mosaicplasty and osteochondral autograft have been used in the hip joint for the management of osteochondral defects on the femoral head, but the clinical results are varied and have thus far been limited to case reports or case series [14, 16]. One possible solution for the treatment of wide articular lesions is the use of osteochondral allografts but some concerns have risen about storage of frozen grafts and also difficulties in availability and management of fresh ones, whose increased risk of infective disease transmission has also to be considered [17]. A closing wedge intertrochanteric osteotomy has been described as an alternative treatment: the impaction area of ​​the femoral head is filled with an autologous bone graft to reconstruct joint congruence. This technique allows to turn the head impaction out of the weight-bearing zone, without restoring the chondral surface [4]. Hip arthroplasty is also a valid therapeutic option for the treatment of wide osteochondral defect on the femoral head, however, it is mainly indicated in elderly patients [18].

In the present case report, a combined biomechanical and biological approach has been used to treat an obturator hip dislocation with a type IVA femoral head fracture according to Brumback classification system with an intraarticular bony fragment. In this case, we decided to fix the largest fragment with two screws and to remove the other small fragments. The residual osteochondral area was replaced by a biomimetic nanostructured osteochondral scaffold with the aim of stimulating better the tissue reconstruction of the damaged articular surface. MaioRegen scaffold has been successfully used to treat osteochondral defects of the knee, mainly in young patients with osteochondritis dissecans or traumatic lesions, [19–22] thanks to its ability to reconstruct both the articular cartilage surface and the subchondral bone layer. Thanks to the scaffold peculiar biomimetic properties, bone marrow stem cell differentiation is promoted and osteochondral tissue restoration achieved [23]. In a serial evaluation of tissue progression over time, MRI images showed a satisfactory appearance at the cartilage tissue and subchondral features steadily improving over time up to midterm follow-up [21].

## Conclusions

The MaioRegen biomimetic osteochondral scaffold could be a promising and safe option for the treatment of traumatic osteochondral lesions of the femoral head, providing good and stable clinical and radiological outcomes at 2-year follow-up. Nonetheless, further studies are needed to better understand the potential of this scaffold in the treatment of osteochondral lesions in the hip joint.

## Data Availability

Not applicable.
